# Effect Modification of Trimethylamine N-Oxide and Lipoprotein Insulin Resistance with Post-Transplantation Diabetes After Liver Transplant

**DOI:** 10.3390/ijms27041959

**Published:** 2026-02-18

**Authors:** Mateo Chvatal-Medina, Yakun Li, Adrian Post, Margery A. Connelly, Han Moshage, Stephan J. L. Bakker, Vincent E. de Meijer, Hans Blokzijl, Robin P. F. Dullaart, on behalf of TransplantLines Investigators

**Affiliations:** 1 Department of Gastroenterology and Hepatology, University Medical Center Groningen, University of Groningen, P.O. Box 30001, 9700 RB Groningen, The Netherlands; y.li01@umcg.nl (Y.L.); a.j.moshage@umcg.nl (H.M.); h.blokzijl@umcg.nl (H.B.); 2 Department of Internal Medicine, Division of Nephrology, University Medical Center Groningen, University of Groningen, P.O. Box 30001, 9700 RB Groningen, The Netherlands; a.post01@umcg.nl (A.P.); s.j.l.bakker@umcg.nl (S.J.L.B.); 3 Labcorp, 100 Perimeter Park, Morrisville, NC 27560, USA; connem5@labcorp.com; 4 Department of Surgery, Division of Hepato-Pancreato-Biliary Surgery and Liver Transplantation, University Medical Center Groningen, University of Groningen, P.O. Box 30001, 9700 RB Groningen, The Netherlands; v.e.de.meijer@umcg.nl; 5 Department of Internal Medicine, Division of Endocrinology, University Medical Center Groningen, University of Groningen, P.O. Box 30001, 9700 RB Groningen, The Netherlands; dull.fam@12move.nl

**Keywords:** post-transplant diabetes mellitus, liver transplantation, trimethylamine N-oxide, lipoprotein insulin resistance score

## Abstract

Post-transplant diabetes mellitus (PTDM) is a common complication after liver transplantation. Trimethylamine N-oxide (TMAO), a microbiota-derived metabolite, has been linked to insulin resistance, but epidemiological findings on type 2 diabetes remain inconsistent. The Lipoprotein Insulin Resistance (LP-IR) score is a nuclear magnetic resonance (NMR)-derived marker of insulin resistance, yet its role in PTDM and interaction with TMAO are unknown. Three hundred sixty-seven (367) liver transplant recipients (LTRs) from the TransplantLines cohort were studied. Baseline TMAO and LP-IR score were quantified by NMR spectroscopy. Incident PTDM was defined by international criteria. Associations were tested using logistic regression and Cox proportional regression analysis. Effect modification was tested with interaction terms. Thirty-one out of 246 LTRs at risk developed PTDM after a median follow-up of 7.1 years. Higher TMAO (OR 2.14, *p* = 0.015) and LP-IR score (OR 1.66, *p* = 0.015) were associated with increased PTDM risk after adjustment for eGFR and immunosuppressant use. A positive interaction was present (*p* = 0.029) with risk amplification when both biomarkers were elevated. TMAO’s association with PTDM was strongest at high LP-IR (90th percentile; OR 3.20, *p* = 0.005), and LP-IR’s association was strongest at high TMAO (90th percentile; OR 2.56, *p* = 0.002). Time-to-event analysis confirmed these findings. The independent and positive interaction of TMAO and LP-IR with PTDM in LTRs would suggest a pro-diabetic action of TMAO that depends on insulin resistance.

## 1. Introduction

Post-transplant diabetes mellitus (PTDM) is the most common metabolic complication following liver transplantation [1,2]. It is defined as the onset of diabetes in a previously non-diabetic liver transplant recipient (LTR), and contributes to increased cardiovascular and infection risk, thereby impacting long-term survival [3,4]. Known risk factors for PTDM in LTR include obesity, age, and male sex, as well as donor- and transplant-related factors including immunosuppressive regimens [5,6]. Despite the potential of some of these factors as predictors for PTDM, there is an unmet need to better delineate metabolic disturbances that impact the development of PTDM [7].

Recently, there has been increasing interest in the relationship between microbiota and metabolic disease, particularly regarding microbiota-derived metabolites, such as trimethylamine N-oxide (TMAO) [8,9]. TMAO is produced through the microbial metabolism of trimethylamine-containing nutrients, such as choline, betaine, and carnitine, which are abundant in most Western diets [10]. Clinically, TMAO is increasingly recognized as a detrimental metabolite because of its association with adverse cardiovascular outcomes in high-risk populations [11,12]. Mechanistic evidence has accumulated that TMAO might impair insulin signaling through protein kinase R-like endoplasmic reticulum kinase (PERK) and increased oxidative stress [13,14]. However, prospective clinical evidence for its association with diabetes mellitus (DM) is inconsistent [15,16,17]. Given the apparent discrepancy between mechanistic evidence and the inconsistent results from epidemiological studies, LTRs, who are at increased risk of incident diabetes due to gut-microbiota alterations from immunosuppression, antibiotic exposure, and diet, may represent an important group to evaluate the effects of TMAO on PTDM [18,19]. Nonetheless, to date, no study has examined the association between TMAO and PTDM in LTRs, nor its interaction with insulin resistance to influence the risk of PTDM.

Surrogate markers of insulin resistance have gained prominence in clinical use. The Lipoprotein Insulin Resistance (LP-IR) score, calculated from six nuclear magnetic resonance (NMR)-derived lipoprotein parameters, is a validated surrogate of both peripheral and hepatic insulin resistance in epidemiological cohorts [11,20,21]. The LP-IR score is closely associated with insulin resistance and glucose metabolism as inferred from its relationship with the homeostasis model of insulin resistance (HOMA-IR), and lipoprotein compositional changes that affect LP-IR scores may reflect an early abnormality in the course of insulin resistance and DM development [20,22,23]. Although high LP-IR scores predict incident DM and cardiovascular disease in the general population, their role in PTDM in LTRs has not yet been characterized [11,23,24].

In this context, we hypothesized that both higher circulating TMAO levels and a higher LP-IR score are associated with PTDM in LTRs and questioned whether each of these markers would modify their putative association with PTDM. Therefore, the aims of this study were (i) to explore the relationship between TMAO and LP-IR scores in a well-characterized cohort of LTRs, (ii) to explore the associations of TMAO and LP-IR with PTDM, and (iii) to assess a potential interaction between TMAO and LP-IR in relation to the risk of developing PTDM.

## 2. Results

### 2.1. Study Population

A total of 367 LTRs were included. Of these, 121 (33.0%) had either a diagnosis of DM before transplantation or developed transient post-transplant hyperglycemia after LT, 215 (58.6%) had no DM at baseline and remained free of PTDM during follow-up, while 31 (8.4%) developed PTDM (Figure S1). Baseline characteristics stratified by glycemic status are shown in Table 1. Patients with baseline DM were older, had higher BMI, and lower eGFR compared with those without DM. Those who developed PTDM had intermediate values for age and BMI but exhibited lipid and lipoprotein profiles that were more similar to the baseline DM group, including higher small LDL-P, larger VLDL size, and lower HDL particle concentrations. Circulating TMAO levels and LP-IR scores were also significantly higher in LTRs with either baseline DM or incident PTDM, compared to recipients who remained free of diabetes.

### 2.2. Correlation Between TMAO and LP-IR

**Figure 1:**
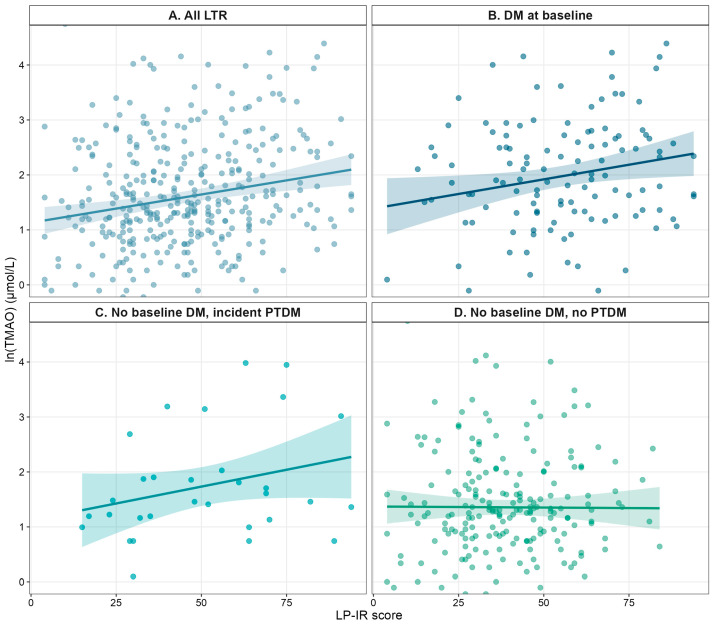
Association between LP-IR score and Circulating TMAO Concentration in Liver Transplant Recipients. Scatterplots with regression lines and 95% CIs are shown for all LTRs (**A**), those with DM at baseline (**B**), those without baseline DM who developed PTDM (**C**), and without baseline DM and no incident PTDM (**D**). Abbreviations: CI: confidence intervals; DM: diabetes mellitus, LP-IR score: lipoprotein insulin resistance score, PTDM: post-transplant diabetes mellitus TMAO: trimethylamine N-oxide (ln-transformed).

In the whole cohort, higher ln-transformed TMAO concentrations were correlated with higher LP-IR scores (β = 0.20, 95% CI 0.09–0.30; *p* < 0.001) as shown in Figure 1. A similar relationship was observed among LTRs with preexisting DM (β = 0.20, 95% CI 0.03–0.38; *p* = 0.025). Also, a positive correlation was found in the subgroup who developed PTDM during follow up (β = 0.49, 95% CI 0.06–0.91; *p* = 0.025). No correlation was present among those without baseline DM who remained free of PTDM (β = −0.02, 95% CI −0.14 to 0.11; *p* = 0.797).

### 2.3. Association Between Post-Transplant Diabetes Mellitus with TMAO and LP-IR

To assess whether TMAO concentrations and LP-IR scores were associated with incident PTDM, logistic regression analyses were performed in the group of 246 LTRs who did not have DM at baseline (Table 2). Over a median follow-up of 7.1 years (95% CI 6.9–7.3 years), 31 LTRs developed PTDM, with a cumulative incidence of 12.6%. These analyses showed that, in crude models (Model 1), both higher TMAO (OR 1.80, 95% CI 1.09–3.00; *p* = 0.023) and higher LP-IR (OR 1.64, 95% CI 1.13–2.39; *p* = 0.009) were associated with increased PTDM risk. In this unadjusted analysis, the interaction term was significant with an OR of 1.76 (95% CI 1.07–2.91; *p* = 0.027), meaning that for each 1-SD increase in LP-IR, the odds for TMAO’s association with PTDM are multiplied by 1.76, and vice versa. Adjustment for age, sex, and BMI (Model 2) attenuated these associations, although the interaction term between TMAO and LP-IR remained statistically significant (OR 1.66, 95% CI 1.00–2.79; *p* = 0.048). In adjusted models accounting for eGFR, use of steroids and tacrolimus (Model 3), the associations of TMAO (OR 2.14, 95% CI 1.17–4.00; *p* = 0.015), LP-IR score (OR 1.66, 95% CI 1.10–2.37; *p* = 0.015) and their interaction term (OR 1.82, 95% CI 1.09–3.17; *p* = 0.029) with PTDM risk were reinforced. The inclusion of the interaction term in the models significantly improved model fit (likelihood ratio test *p* = 0.034). Time-to-event analyses showed consistent associations of TMAO, LP-IR score and their interaction term with PTDM, with similar behavior in unadjusted and adjusted models as those in the logistic regression models (Table 3). To further confirm this association, in Ridge-penalized regression models the association with PTDM was maintained for both markers and their interaction term, and all associations became significant in models adjusting for age, sex, and BMI (Table S1).

### 2.4. The Interaction Between TMAO and LP-IR for the Development of Post-Transplant Diabetes

**Figure 2:**
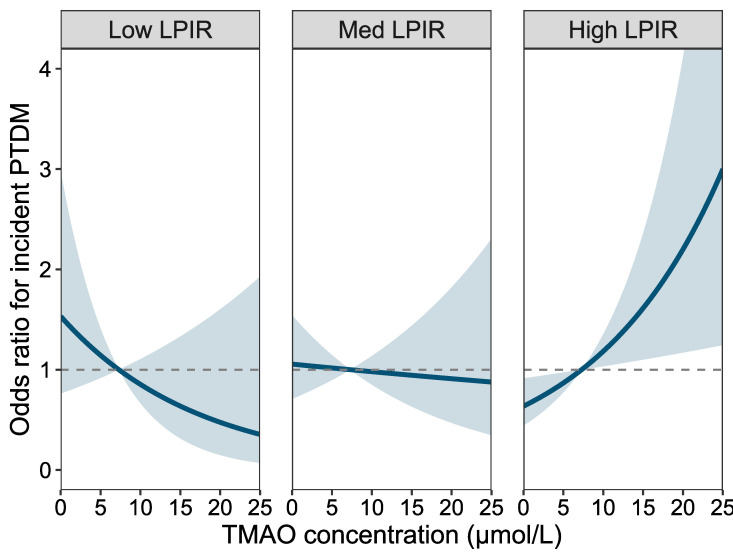
Effect modification in LP-IR score on the association between trimethylamine N-oxide (TMAO) on the Risk of Post-Transplant Diabetes Mellitus Logistic regression analysis model with TMAO (centered on the mean) and LP-IR score, without adjustment for covariates, corresponding to model 1 in Table 2. Odds ratios represent the effect of TMAO concentration on the risk of PTDM at the 10th (low), 50th (median), and 90th (high) percentiles of the LP-IR score. Dashed lines represent the null-effect, and the blue continuous line represents the regression line in each model. Shaded areas denote 95% confidence intervals. Abbreviations: LP-IR score: lipoprotein insulin resistance score, Med: median, PTDM: post-transplant diabetes mellitus, TMAO: trimethylamine N-oxide.

**Figure 3:**
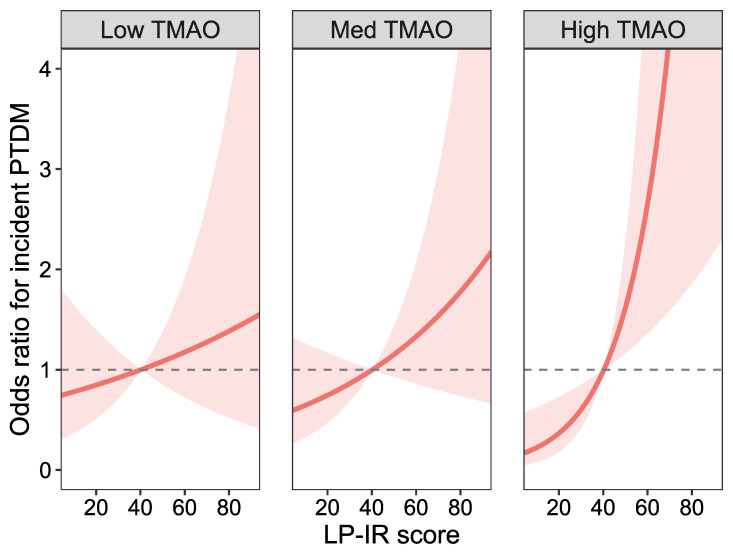
Effect modification in TMAO on the association between LP-IR score and PTDM Logistic regression analysis model with LP-IR score (centered on the mean) and TMAO, without adjustment for covariates, corresponding to model 2 in Table 2. Odds ratios represent the effect of LP-IR score on the risk of PTDM at the 10th, 50th, and 90th percentiles of TMAO concentration. Dashed lines represent the null-effect, and the blue continuous line represents the regression line in each model. Shaded areas denote 95% confidence intervals. Abbreviations: LP-IR score: lipoprotein insulin resistance score, Med: median, PTDM: post-transplant diabetes mellitus, TMAO: trimethylamine N-oxide.

Given the presence of a statistically significant interaction in all models, we next conducted effect modification analyses (Table 4; Figure 2 and Figure 3). When stratified by LP-IR score, the association between TMAO and PTDM was strongest among recipients with a high LP-IR score (90th percentile; OR 3.20, 95% CI 1.46–7.03; *p* = 0.005), whereas no significant associations were observed in the low or medium LP-IR strata. Conversely, when stratified by TMAO levels, the association between LP-IR and PTDM was strongest in the high TMAO stratum (90th percentile; OR 2.56, 95% CI 1.41–4.64; *p* = 0.002), and similarly, no association was seen in low and medium TMAO strata. The effect modification was corroborated in Cox regression models, in which the association between markers with PTDM was only seen in the highest percentiles of each biomarker (Table S2). Graphical representations of these interactions (Figure 2 and Figure 3) illustrate the amplification of PTDM risk when both TMAO and LP-IR are elevated.

## 3. Discussion

In the present prospective study, among 246 LTRs without DM at baseline, 31 patients (12.6%) developed PTDM over a median follow-up of 7.1 years. Our findings demonstrate that (i) baseline TMAO and the LP-IR score are positively correlated among LTRs with pre-existing diabetes as well as among LTRs who developed PTDM, but not among LTRs who stayed free of PTDM, (ii) higher TMAO and LP-IR scores were each associated with increased risk of PTDM, and (iii) there is a significant interaction between TMAO and LP-IR for the risk of developing PTDM, with excess risk concentrated among recipients in whom both biomarkers were elevated. Likewise, our study shows risk amplification at jointly high levels, where TMAO’s association was strongest at the 90th percentile of LP-IR, and LP-IR’s association with PTDM was strongest at the 90th percentile of TMAO. Combined, our findings suggest that TMAO and lipoprotein alterations captured by the LP-IR score are subject to risk amplification in subjects with PTDM.

Our study should be regarded as hypothesis-generating. Nonetheless, several pathways could conceivably be implicated in the association between TMAO and insulin resistance. Firstly, experimental models demonstrate that TMAO binds to and activates PERK, driving PERK-eIF2α and inducing forkhead box protein O1 (FoxO1), thereby stimulating hepatic gluconeogenesis and contributing to the establishment of hepatic insulin resistance [13,24]. These effects can be reversed through inhibition or knockdown of PERK or attenuated through flavin monooxygenase 3 (FMO3) inhibition [13]. Since FMO3 is responsible for the metabolism of trimethylamine (TMA) into TMAO in the liver, its inhibition results in lower TMAO concentration [25]. Because insulin normally suppresses FMO3, both insulin resistance and insulin deficiency result in FMO3 overactivity and higher TMAO production, contributing to a vicious cycle [13,24,26]. Secondly, at higher concentrations, TMAO might play a role in β-cell dysfunction by disrupting Ca^2+^ regulation through autophagic degradation of Sarco/Endoplasmic Reticulum Calcium ATPase 2 [27]. This leads to lower oxidative phosphorylation capacity and ATP synthesis in the mitochondria, which, upon long exposure, promotes ER stress through unfolded protein response, and eventually apoptosis in β-cells [26,28]. Thirdly, TMAO exposure might contribute to the low-grade chronic inflammation seen in patients with metabolic dysfunction. TMAO activates the NLRP3 inflammasome in pancreatic islets, leading to IL-1β release, which not only impairs insulin secretion but also promotes peripheral insulin resistance [29]. Furthermore, TMAO stimulates NF-κB, which contributes to a proinflammatory milieu through TNF-α and IL-6 [30].

These pathways align with our current observation that TMAO is more strongly associated with PTDM in the context of a higher LP-IR score, as a lipoprotein proxy of insulin resistance, whereas the LP-IR score is more strongly associated with PTDM when circulating TMAO levels are elevated. Although this approach is merely exploratory, our findings suggest that insulin resistance might condition the diabetogenic potential of TMAO, such that an association becomes evident only at higher degrees of insulin resistance. The context dependence partly reconciles the inconsistent associations of TMAO with DM in prior epidemiological studies, which generally did not stratify by insulin resistance phenotypes [15,16,17,31,32]. In transplantation, this effect modification is likely accentuated given that immunosuppressants and antibiotics alter the gut microbiome and its metabolite production, and kidney function modulates circulating TMAO [33,34]. Corticosteroids and mTOR inhibitors have both been implicated in promoting insulin resistance and β-cell dysfunction through mechanisms including enhanced gluconeogenesis, lipotoxicity, and impaired insulin signaling [5,19,35]. Although we adjusted for eGFR and immunosuppressants, residual variability in drug exposure and gut microbial composition cannot be excluded. Furthermore, while no direct evidence exists that steroids modify the association between TMAO and incident diabetes, their known effects on gut microbiota and host metabolism make such effect modification biologically plausible [36,37]. Of note, although the role of β-cell dysfunction is mechanistically reasonable, we cannot draw conclusions on it, as no reliable surrogate marker was available.

From a clinical perspective, combining TMAO with LP-IR, two NMR-derived measurements obtained from a single fasting plasma sample, could refine PTDM risk stratification beyond traditional clinical predictors. A combined approach could help target intensified monitoring and early preventive strategies for those at highest risk. Notably, LP-IR is an established marker for insulin resistance, incident diabetes and cardiovascular disease in population studies, and though TMAO showed conflicting evidence previously, our findings might support its usefulness, given that it is also easily quantified by clinical NMR or LC-MS platforms. However, given that this study is hypothesis-generating, further studies are needed to show the incremental predictive value, discrimination, and calibration of these as formal markers for risk stratification of PTDM.

This study has several strengths and limitations. It is the first to evaluate comprehensively the association of TMAO and LP-IR with PTDM in LTRs, and the first to assess the interaction of these two biomarkers with incident diabetes in this patient category. Furthermore, the measurements of TMAO and the lipoprotein components used for calculating LP-IR were done using well-validated NMR spectroscopy assays. An additional strength that might mitigate the limited number of events that took place in the study period, was the corroboration of a potentially true biological phenomenon through both logistic and Cox regression models, which accounted for adjustment for multiple clinical covariates, as well as the use of Ridge-penalized models. Our study also has limitations. Firstly, LTRs were included at various time points after LT, which can result in disparities when comparing their baseline levels. The retrospective set-up of the Transplantlines Biobank and Cohort Study led us to primarily use logistic regression analysis rather than time-to-event analysis to analyze the impact of TMAO and the LP-IR score on PTDM, although Cox regression models were also included as a secondary set-up to reveal the robustness of the findings. Secondly, baseline plasma insulin was not measured in the cohort, precluding direct comparisons with insulin resistance indices like HOMA-IR in their association with P2DM. Notably, LP-IR has been proposed to obviate the necessity to measure insulin and document insulin resistance, as has been previously validated [11,20,21,38]. Thirdly, 31 PTDM events accrued, which may limit statistical power. For this reason, we adjusted for potential confounders in two separate models, each including 3 variables. Notably, statin use was not included in the confounder set as they were not often used in LTRs who developed PTDM than in those who did not. This seems relevant as statin use may give rise to deterioration in glucose tolerance [39]. Further, although adjustment for immunosuppressing medication was performed, dosage information was not specifically available. Therefore, residual confounding by treatment intensity cannot be excluded. Fourthly, since this is an observational study, inference to causality is precluded. Finally, given the predominant North European origin of patients in our study, the findings cannot be extrapolated to other ethnicities.

## 4. Materials and Methods

### 4.1. Study Population and Design

We conducted a prospective cohort study within the TransplantLines Biobank and Cohort Study (NCT03272841), a large ongoing observational study at the University Medical Center Groningen (UMCG), the Netherlands, that includes all types of solid organ transplant recipients [40,41]. Biomaterials and extensive demographic, clinical, and lifestyle data were retrieved. Exclusion criteria were: inability to provide informed consent due to language or cognitive barriers, re-transplantation, and missing data on primary laboratory measurements (TMAO or LP-IR score components). For the present study, we included liver transplant recipients LTRs with available data on plasma TMAO concentrations, LP-IR score, as defined below, and diabetes status. Data collection for baseline variables in LTRs extended through June 2021, and follow-up for outcomes was completed through 1 February 2025.

The study protocol was approved by the Institutional Review Board of the UMCG (METc 2014/077) and was conducted in accordance with the Declaration of Helsinki. This study was reported in accordance with the STROBE guidelines. All participants provided written informed consent.

### 4.2. Baseline Data Collection

At inclusion, participants underwent standardized outpatient visits consisting of structured interviews, physical examination, and laboratory sampling. Demographic and clinical data included age, sex, body mass index (BMI), blood pressure, immunosuppressive regimen, and comorbidities. BMI was calculated as weight (kg) divided by height squared (m^2^). Hypertension was defined as (i) systolic blood pressure ≥ 140 mmHg, (ii) diastolic blood pressure ≥ 90 mmHg, or (iii) current antihypertensive treatment. Diabetes at baseline was defined by (i) fasting plasma glucose ≥ 126 mg/dL (7.0 mmol/L), (ii) random plasma glucose ≥ 200 mg/dL (11.1 mmol/L), (iii) glycated hemoglobin (HbA1c) ≥ 6.5% (48 mmol/mol), or (iv) use of glucose-lowering drugs. Estimated glomerular filtration rate (eGFR) was calculated using the CKD-EPI creatinine equation [42]. Data on steroid use, calcineurin inhibitor use (tacrolimus and cyclosporine), and mTOR or antiproliferative therapy were extracted from medical records.

### 4.3. Outcome Definition

The primary outcome was incident PTDM after liver transplantation. PTDM was defined according to international criteria [43]: (i) fasting plasma glucose ≥ 126 mg/dL (7.0 mmol/L), (ii) random plasma glucose ≥ 200 mg/dL (11.1 mmol/L), (iii) HbA1c ≥ 6.5% (48 mmol/mol), or (iv) initiation of glucose-lowering therapy, whichever occurred first after baseline (i.e., time of blood sampling). Cases of transient posttransplant hyperglycemia, defined as those with ≥2 times fasting plasma glucose values ≥ 126 mg/dL within the first 90 days post-LT, as well as those with altered fasting plasma glucose or HbA1c values within the first year after LT, were excluded. Outcomes were ascertained from electronic medical records and validated by treating hepatologists. Follow-up accrued from the baseline visits until the earliest of PTDM diagnosis, death, last available clinical contact, or 1 February 2025.

### 4.4. Laboratory Measurements

Venous blood was collected after an overnight fast of at least 10 h. EDTA plasma samples were processed within 2 h and stored at −70 °C until shipped for analysis to Labcorp Inc. (Morrisville, NC, USA). NMR spectra were collected from plasma samples using the Vantera^®^ Clinical Analyzer (Labcorp Inc., Raleigh, NC, USA) [44]. TMAO was quantified from one-dimensional proton (1H) Carr-Purcell-Meiboom-Gill (CPMG) spectra using a deconvolution assay as previously described [45]. The TMAO assay has intra- and inter-assay coefficients of variation (CV%) of 4.3 and 9.8%, respectively [46]. Very-low-density lipoprotein (VLDL), low-density lipoprotein (LDL), and high-density lipoprotein (HDL) particle concentrations, subfractions, and mean particle sizes were quantified from the amplitudes of their spectroscopically distinct lipid methyl group NMR signals using the standard lipoprotein spectral acquisition [23]. Total particle concentrations for VLDL, LDL, and HDL were calculated as the sum of their respective subclasses. Intra-assay CVs for lipoprotein parameters were 11.0% for VLDL concentration, 4.1% for LDL concentration, 2.0% for HDL concentration, and 6.6–27.9% for subfractions. Mean VLDL, LDL, and HDL sizes were calculated as weighted averages of subclass diameters. The LP-IR score was derived from six NMR-measured lipoprotein variables: the weighted average sizes of VLDL, LDL, and HDL combined with the concentrations of large VLDL, small LDL, and large HDL particles [23]. LP-IR scores range from 0 to 100, with higher values indicating greater insulin resistance. The LP-IR scores were calculated using the LP3 algorithm designed by Labcorp [20]. Plasma glucose, HbA1c, and serum creatinine were measured at the UMCG Department of Laboratory Medicine with standardized quality-controlled assays.

### 4.5. Statistical Analysis

All analyses were conducted using R (version 4.4.1, R Foundation for Statistical Computing, Vienna, Austria). A two-sided *p*-value < 0.05 was considered statistically significant. Baseline characteristics were summarized as median [interquartile range], or *n* (%), where appropriate. Group differences were assessed using χ^2^ for categorical variables, and Kruskal–Wallis tests for continuous variables. Liver transplant recipients with pre-transplant DM or early PTDM were excluded from prospective analyses. Cross-sectional associations between plasma TMAO concentrations and LP-IR scores were examined using linear regression models. Analyses were performed in the full cohort and stratified by baseline diabetes status and by the development of incident PTDM. The results are reported as standardized β coefficients (β) with 95% confidence intervals. Associations of plasma TMAO (ln-transformed) and LP-IR score with incident PTDM were examined using logistic regression models and Cox regression models (per 1-SD increases), with unadjusted models and two adjustment sets: (i) adjusting for age, sex, and BMI, and (ii) adjusting for eGFR, steroid use, and use of calcineurin inhibitors. The results for logistic and Cox regression models are reported as odds ratios (OR) and hazard ratios (HR), respectively, with 95% confidence intervals (CI). Linearity of continuous exposures was confirmed by comparing linear models with restricted cubic spline models using the likelihood ratio test. Multiplicative interaction terms were added to the models, and likelihood ratio tests compared models with and without interaction terms. For significant interactions, effect modification was further examined by calculating marker-specific ORs at 10th, 50th, and 90th percentiles for LP-IR score and TMAO with parametric bootstrapping to generate CIs. For Cox regression analyses, the proportional hazards assumption was tested with Schoenfeld residuals and was not violated.

## 5. Conclusions

In this prospective cohort of LTRs, higher baseline TMAO concentrations and LP-IR scores were each associated with an increased risk of PTDM. Importantly, joint elevation identified recipients at particularly higher risk, which suggests a potential interaction between gut microbiota-derived metabolites and lipoprotein alterations linked to insulin resistance. Combined, these findings suggest that TMAO’s diabetogenic potential may be conditional on the degree of insulin resistance, which could partly reconcile inconsistencies from previous epidemiological studies. From a clinical perspective, combining TMAO and LP-IR, two NMR-derived measures obtained from a single fasting sample, may better provide early risk stratification for PTDM beyond traditional predictors. Future studies are warranted to validate these results, explore the modifying role of immunosuppressive regimens and kidney function, and determine whether this combination can guide personalized immunosuppression in LTRs.

## Figures and Tables

**Table 1:** Baseline characteristics of liver transplant recipients included in this study.

Variable		All LTRs (*n* = 367)	DM at Baseline (*n* = 121)	No DM at Baseline, Incident PTDM (*n* = 31)	No DM at Baseline, No Incident PTDM (*n* = 215)	ANOVA *p*-Value
Age (years)		59.0 [47.0–67.0]	65.0 [56.0–68.0]	62.0 [56.5–67.5]	55.0 [42.0–64.0]	<0.001
Sex (%)	Male	215 (58.6)	21 (67.7)	83 (68.6)	111 (51.6)	0.005
	Female	152 (41.4)	10 (32.3)	38 (31.4)	104 (48.4)	
Systolic BP (mmHg)		130 [120–142]	133 [120–146]	135 [120–141]	127 [120–139]	0.092
Diastolic BP (mmHg)		79 [73–87]	77 [72–86]	78 [72–85]	80 [74–87]	0.270
BMI (kg/m^2^)		25.9 [23.0–29.5]	27.8 [24.1–31.3]	27.1 [24.8–30.1]	25.1 [22.6–27.4]	<0.001
eGFR (mL/min/1.73 m^2^)		77.9 [61.1–97.5]	71.7 [54.2–85.3]	77.5 [61.9–86.1]	84.4 [65.6–100.7]	<0.001
Use of statins (%)		84 (22.9%)	50 (41.3%)	3 (9.7%)	31 (14.4%)	<0.001
Use of Steroids (%)		166 (45.4)	52 (43.3)	12 (38.7)	102 (47.4)	0.580
Use of mTOR inhibitors (%)		46 (12.5)	22 (18.2)	5 (16.1)	19 (8.8)	0.033
Use of calcineurin inhibitors (%)	None	106 (28.9)	34 (28.1)	8 (25.8)	64 (29.8)	0.899
	Tacrolimus	227 (61.9)	78 (64.5)	20 (64.5)	129 (60.0)	
	Cyclosporine	34 (9.3)	9 (7.4)	3 (9.7)	22 (10.2)	
Use of antiproliferative drugs (%)	None	179 (48.8)	59 (48.8)	16 (51.6)	104 (48.4)	0.058
	Azathioprine	83 (22.6)	20 (16.5)	4 (12.9)	59 (27.4)	
	Mycophenolate	105 (28.6)	42 (34.7)	11 (35.5)	52 (24.2)	
Glucose (mg/dL)		98.0 [87.5–116.5]	131.0 [103.0–169.0]	100.0 [93.0–111.0]	92.0 [83.5–99.5]	<0.001
Total cholesterol (mmol/L)		4.2 [3.6–4.8]	4.0 [3.6–4.7]	4.3 [4.0–5.1]	4.2 [3.7–4.8]	0.041
HDL cholesterol (mmol/L)		1.3 [1.1–1.7]	1.1 [1.0–1.5]	1.2 [1.0–1.6]	1.4 [1.2–1.7]	<0.001
LP-IR score		44.0 [30.0–59.0]	56.0 [41.0–71.0]	47.0 [30.0–66.5]	37.0 [28.0–50.0]	<0.001
Large VLDL-P (nmol/L)		3.9 [2.2–7.3]	5.9 [3.3–10.1]	3.9 [2.0–8.2]	3.0 [1.9–5.2]	<0.001
Small LDL-P (nmol/L)		506.0 [84.0–664.0]	600.0 [392.0–741.0]	579.0 [366.5–767.5]	430.0 [73.0–609.5]	<0.001
Large HDL-P (nmol/L)		8.0 [5.2–11.1]	5.9 [4.4–9.9]	7.0 [4.2–10.6]	9.0 [6.4–11.6]	<0.001
VLDL size (nm)		51.2 [47.1–56.0]	53.9 [48.7–58.6]	51.4 [45.4–58.3]	49.8 [46.5–54.1]	<0.001
LDL size (nm)		20.9 [20.2–21.3]	20.3 [20.0–21.0]	21.0 [20.6–21.2]	21.0 [20.5–21.4]	<0.001
HDL size (nm)		9.6 [9.1–10.3]	9.2 [8.9–9.9]	9.5 [8.9–10.1]	9.8 [9.4–10.3]	<0.001
TMAO (μmol/L)		4.3 [2.6–9.6]	6.8 [3.8–14.3]	4.4 [3.3–7.2]	3.6 [2.2–6.6]	<0.001

Data are expressed in median [interquartile range], and in numbers (percentages) for categorical variables. *p*-values represent global comparisons across the three groups (Kruskal–Wallis test for continuous variables and Chi-squared test for categorical variables). Abbreviations: ANOVA: analysis of variance, BP: blood pressure, BMI: body mass index, DM: Diabetes mellitus, eGFR: estimated glomerular filtration rate, HDL: high density lipoprotein, LDL: low density lipoprotein, LP-IR: lipoprotein insulin resistance score, LTRs: liver transplant recipients, mTOR: mechanistic target of rapamycin, PTDM: post-transplant diabetes mellitus, TMAO: trimethylamine N-oxide, VLDL: very low density lipoproteins.

**Table 2:** Logistic regression analysis showing association between trimethylamine N-oxide (TMAO), lipoprotein insulin resistance (LP-IR) score, and their interactions with post-transplant diabetes mellitus.

Model	TMAO		LP-IR Score		TMAO: LP-IR Score Interaction	
	OR [95% CI]	*p*-Value	OR [95% CI]	*p*-Value	OR [95% CI]	*p*-Value
Model 1	**1.80 [1.09–3.00]**	**0.023**	**1.64 [1.13–2.39]**	**0.009**	**1.76 [1.07–2.91]**	**0.027**
Model 2	1.47 [0.86–2.64]	0.187	1.33 [0.89–2.01]	0.160	**1.66 [1.00–2.79]**	**0.048**
Model 3	**2.14 [1.17–4.00]**	**0.015**	**1.66 [1.10–2.37]**	**0.015**	**1.82 [1.09–3.17]**	**0.029**

Logistic regression analysis showing the association between TMAO, LP-IR score, and PTDM. Model 1 represents the crude model. Model 2 represents the adjusted set, which includes age, sex, and BMI. Model 3 represents the adjusted set including eGFR, steroid use, and tacrolimus use. Statistically significant odds ratios in each model are shown in bold. TMAO is ln-transformed, then standardized; LP-IR score is standardized. BMI: body-mass index, CI: confidence interval, eGFR: estimated glomerular filtration rate, LP-IR score: lipoprotein insulin resistance score, OR: odds-ratio, TMAO: trimethylamine N-oxide.

**Table 3:** Cox regression analysis showing association between trimethylamine N-oxide (TMAO), lipoprotein insulin resistance (LP-IR) score, and their interactions with post-transplant diabetes mellitus.

Model	TMAO		LP-IR Score			TMAO: LP-IR Score Interaction
	HR [95% CI]	*p*-Value	HR [95% CI]	*p*-Value	HR [95% CI]	*p*-Value
Model 1	**1.72 [1.03–2.67]**	**0.036**	**1.58 [1.12–2.26]**	**0.026**	**1.54 [1.04–2.35]**	**0.032**
Model 2	1.41 [0.79–2.38]	0.227	1.26 [0.89–2.01]	0.176	**1.60 [1.02–2.62]**	**0.041**
Model 3	**1.99 [1.12–3.85]**	**0.021**	**1.61 [1.09–2.27]**	**0.024**	**1.52 [1.04–3.06]**	**0.029**

Cox regression analysis showing the association between TMAO, LP-IR score, and PTDM. Model 1 represents the unadjusted model. Model 2 represents the model adjusted for age, sex, and BMI. Model 3 represents the adjusted set including eGFR, steroid use, and tacrolimus use. Statistically significant hazard ratios in each model are shown in bold. TMAO is ln-transformed, then standardized; LP-IR score is standardized. BMI: body-mass index, CI: confidence interval, eGFR: estimated glomerular filtration rate, LP-IR score: lipoprotein insulin resistance score, HR: hazard ratio, TMAO: trimethylamine N-oxide.

**Table 4:** Effect modifications of lipoprotein insulin resistance (LP-IR) score and trimethylamine N-oxide (TMAO) on the risk of post-transplant diabetes mellitus.

Effect (Per-SD)	Moderator Stratum	OR [95% CI]	*p*-Value
**Model 1:** **TMAO** across LP-IR score strata	Low	0.76 [0.34–1.71]	0.516
	Medium	1.40 [0.85–2.33]	0.193
	**High**	**3.20 [1.46–7.03]**	**0.005**
**Model 2:** **LP-IR** score across TMAO strata	Low	0.90 [0.48–1.69]	0.744
	Medium	1.38 [0.92–2.08]	0.119
	**High**	**2.56 [1.41–4.64]**	**0.002**

Model 1 focuses on TMAO as the main variable and LP-IR score as an effect modifier, while Model 2 focuses on LP-IR score as the main variable and TMAO as an effect modifier. ORs quantify the change in odds of incident PTDM per 1 SD increase in the main marker per model, estimated from a logistic model with centered variables, both the main and the modifier of effect. “Low/Medium/High” denote the moderator at its 10th/50th/90th percentile cut-offs, respectively. Statistically significant odds ratios in each model are shown in bold. TMAO is ln-transformed. Abbreviations: LP-IR score: lipoprotein insulin resistance score, OR: odds ratio, SD: standard deviation, TMAO: trimethylamine N-oxide.

## Data Availability

The datasets generated and analyzed in this work are not publicly available due to privacy restrictions. Data may be obtained from the corresponding author upon reasonable request and with approval from the TransplantLines committee.

## Supplementary Materials

The following supporting information can be downloaded at: https://www.mdpi.com/article/10.3390/ijms27041959/s1.

## Abbreviations

The following abbreviations are used in this manuscript:

ANOVA	Analysis of variance
Β	Standardized regression coefficient
BMI	Body mass index
BP	Blood pressure
CI	Confidence interval
CKD-EPI	Chronic Kidney Disease Epidemiology Collaboration
DM	Diabetes mellitus
eGFR	Estimated glomerular filtration rate
FMO3	Flavin monooxygenase 3
FoxO1	Forkhead box protein O1
HbA1c	Glycated hemoglobin
HDL	High-density lipoprotein
IL	Interleukin
IRB	Institutional Review Board
LC-MS	Liquid chromatography-mass spectrometry
LDL	Low-density lipoprotein
LP-IR	Lipoprotein insulin resistance score
LTR	Liver transplant recipient
mTOR	Mechanistic target of rapamycin
NLRP3	NOD-like receptor family pyrin domain-containing 3
NMR	Nuclear magnetic resonance
OR	Odds-ratio
OXPHOS	Oxidative phosphorylation
PERK	Protein kinase R-like endoplasmic reticulum kinase
PTDM	Post-transplant diabetes mellitus
R^2^	Coefficient of determination
SD	Standard deviation
SERCA2a	Sarco/endoplasmic reticulum Ca^2+^-ATPase 2a
TMA	Trimethylamine
TMAO	Trimethylamine N-oxide
TNF	Tumor necrosis factor
UMCG	University Medical Center Groningen
VLDL	Very-low-density lipoprotein
