# Supplementary material for: Effect Modification of Trimethylamine N-Oxide and Lipoprotein Insulin Resistance with Post-Transplantation Diabetes After Liver Transplant
Source: Int J Mol Sci. 2026 Feb 18;27(4):1959. doi: 10.3390/ijms27041959 (PMC12940386; doi:10.3390/ijms27041959)
Supplement: Supplementary file 1 [file ijms-27-01959-s001.zip › ijms-4136088-supplementary.pdf]

**Supplementary Material: Synergism of Trimethylamine N-oxide and Lipoprotein Insulin Resistance with Post-Transplantation Diabetes after Liver Transplant**

Mateo Chvatal-Medina[1*], Yakun Li[1], Adrian Post[2], Margery A. Connelly[3], Han Moshage[1], Stephan J. L. Bakker[2], Vincent E. de Meijer[4], Hans Blokzijl[1], Robin P.F. Dullaart[5], on behalf of TransplantLines investigators

**Contents:**

1. List of Investigators
2. Supplementary Figure 1

**List of Investigators:**

Coby Annema, Stephan J L Bakker, Stefan P Berger, Hans Blokzijl, Frank A J A Bodewes, Marieke T de Boer, Kevin Damman, Martin H de Borst, Belle Dielwart, Arjan Diepstra, Gerard Dijkstra, Caecilia S E Doorenbos, Rianne M Douwes, Michele F Eisenga, Michiel E Erasmus, C Tji Gan, Antonio W Gomes Neto, Eelko Hak, Bouke G Hepkema, Jip Jonker, Frank Klont, Tim J Knobbe, Daan Kremer, Henri G D Leuvenink, Willem S Lexmond, Vincent E de Meijer, Hubert G M Niesters, Gertrude J Nieuwenhuijs-Moeke, L Joost van Pelt, Robert A Pol, Anna M Posthumus, Adelita V Ranchor, Jan Stephan F Sanders, Marion J Siebelink, Riemer J H J A Slart, J Casper Swarte, Daan J Touw, Marius C van den Heuvel, Coretta van Leer-Buter, Marco van Londen, Charlotte A te Velde Keyzer, Erik A M Verschuuren, Michel J Vos, Rinse K Weersma

**Figure S1:** Flow-chart of Patient Distribution According to Previous Diagnosis of Diabetes Mellitus and Post-Transplant Diabetes Mellitus

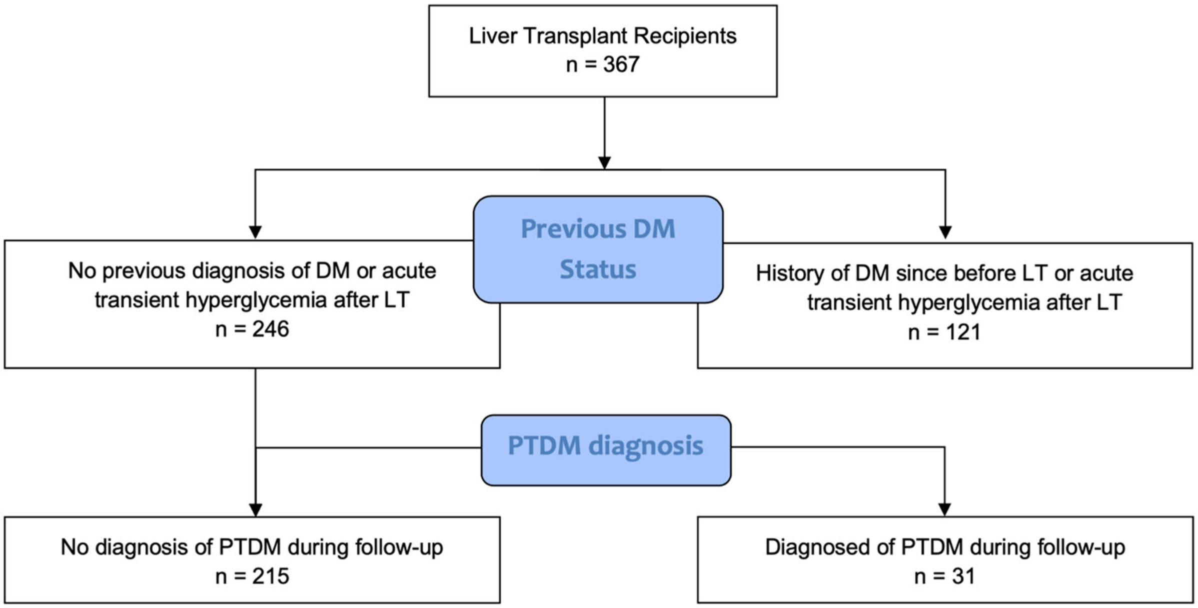


DM: diabetes mellitus, PTDM: post-transplant diabetes mellitus

**Table S1:** Ridge-Penalized Logistic and Cox Regression Analyses Showing Association between Trimethylamine N-oxide (TMAO), Lipoprotein Insulin Resistance (LP-IR) score, and their Interactions with Post-Transplant Diabetes Mellitus.

| Model | TMAO | | LP-IR score | | TMAO: LP-IR score Interaction | |
|---|---|---|---|---|---|---|
| | OR [95% CI] | p-value | OR [95% CI] | p-value | OR [95% CI] | p-value |
| **Model 1** | **1.63 [1.20–2.35]** | **0.009** | **1.31 [1.16–1.83]** | **0.018** | **1.44 [1.08–1.94]** | **0.017** |
| **Model 2** | **1.38 [1.07–1.77]** | **0.019** | **1.18 [1.03–1.49]** | **0.033** | **1.30 [1.00–1.66]** | **0.038** |
| **Model 3** | **1.63 [1.12–2.28]** | **0.007** | **1.45 [1.19–1.84]** | **0.010** | **1.47 [1.04–1.93]** | **0.013** |
| | HR [95% CI] | p-value | HR [95% CI] | p-value | HR [95% CI] | p-value |

| | | | | | | |
|---|---|---|---|---|---|---|
| **Model 1** | **1.55 [1.08–2.05]** | **0.007** | **1.25 [1.06–1.60]** | **0.039** | **1.30 [1.01–1.63]** | **0.028** |
| **Model 2** | 1.31 [1.03–1.59] | 0.011 | 1.14 [0.96–1.37] | 0.119 | 1.20 [0.96–1.46] | 0.085 |
| **Model 3** | **1.62 [1.15–2.30]** | **0.009** | **1.42 [1.04–2.09]** | **0.049** | **1.30 [1.02–1.62]** | **0.045** |

Cox regression analysis showing the association between TMAO, LP-IR score, and PTDM. Model 1 represents the unadjusted model. Model 2 represents the adjusted set for age, sex, and BMI. Model 3 represents the adjusted set including eGFR, steroid use, and tacrolimus use. Statistically significant hazard ratios in each model are shown in bold. TMAO is ln-transformed, then standardized; LP-IR score is standardized. BMI: body-mass index, CI: confidence interval, eGFR: estimated glomerular filtration rate, LP-IR score: lipoprotein insulin resistance score, HR: hazard ratio, TMAO: trimethylamine N-oxide.

**Table S2:** Cox Regression Analysis Showing the Effect Modification of Lipoprotein Insulin Resistance (LP-IR) score and Trimethylamine N-oxide (TMAO) on the Risk of Post-Transplant Diabetes Mellitus.

| Effect (per-SD) | Moderator stratum | HR [95% CI] | p-value |
|---|---|---|---|
| Model 1: TMAO across LP-IR score strata | Low | 0.72 [0.26–1.64] | 0.702 |
| | Medium | 1.35 [0.81–2.27] | 0.208 |
| | **High** | **3.07 [1.33–5.89]** | **0.008** |
| Model 2: LP-IR score across TMAO strata | Low | 0.78 [0.46–1.67] | 0.739 |
| | Medium | 1.34 [0.89–1.94] | 0.231 |
| | **High** | **2.45 [1.31–4.58]** | **0.004** |

Model 1 focuses on TMAO as the main variable and LP-IR score as an effect modifier, while Model 2 focuses on LP-IR score as the main variable and TMAO as an effect modifier. HRs quantify the change in hazard of incident PTDM per 1 SD increase in the main marker per

model, estimated from a Cox regression model with centered variables, both the main and the modifier of effect. “Low/Medium/High” denote the moderator at its 10th/50th/90th percentile cut-offs, respectively. TMAO is ln-transformed. Abbreviations: LP-IR score: lipoprotein insulin resistance score, HR: hazard ratio, SD: standard deviation, TMAO: trimethylamine N-oxide.
